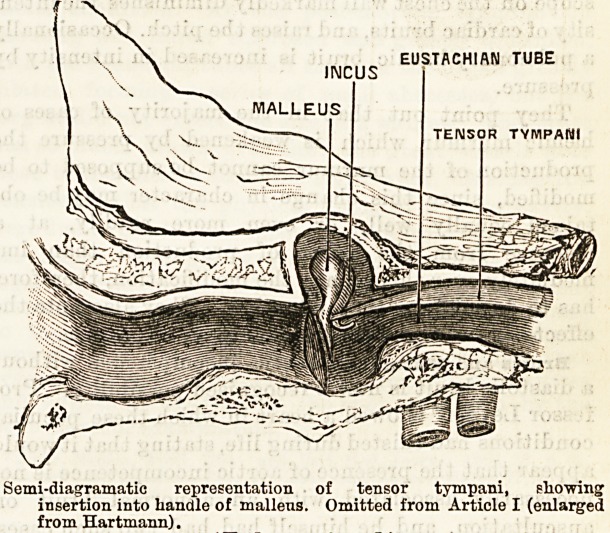# Chronic Catarrh of the Middle Ear —IV

**Published:** 1894-09-15

**Authors:** 


### MEDICAL PROGRESS AND HOSPITAL CLINICS.

[*The Editor will be glad to receive offers of co-operation and contributions from members of the profession. All letters should be addressed to* THE EDITOR, THE LODGE, PORCHESTER SQUARE, LONDON, W.]

### CHRONIC CATARRH OF THE MIDDLE EAR.—IV.

**Inspection of the Drum Membrane.**—Inspection of the drum membrane is best taken up next, in order to see the condition of parts before inflation has been practised. To get a good view of the membrane, a forehead mirror having an oval aperture and a focus of about eight inches, should be worked with from the first, and the surgeon may conveniently accustom himself to wear the mirror so as to see through the aperture with his right eye. The spectacle frame will be found to have many advantages over the forehead band, but tastes differ in this respect. In any case the mirror should have free lateral movement. The best form of illumination (sunlight, of course, excepted, which is rarely available), is that furnished by an argand-burner, with bull's eye if preferred, adapted to a parallel gas bracket. The horizontal arm of the latter is all the better for having an extra joint in the centre, for the purpose of getting a more accurate adjustment of the light, especially if a condenser is used. The heat given off by the metal chimney of the latter is trying to some patients, but this may be partially obviated by enclosing it in an outer case whitened inside like the chimney and separated from it by about one inch space all round. The light should be placed to the (surgeon's) right of the patient's head, and on a level with his ear. With regard to the aural speculum, the chief point is to choose one, whatever the pattern may be, and whether of silver or vulcanite, the tube of which is adapted to the transverse diameter of the meatus under examination. For this purpose it is necessary to have a set of three or four sizes, out of which one can easily be found which will fit exactly. The tubes of the most useful forms (which are more or less funnel-shaped) are oval in transverse section, and therefore require rotating slightly towards the middle line of the face, because the vertical axis of the cartilaginous meatus has an obliquity in this direction. The patient's head must be deliberately placed in such a position as to allow the various segments of the membrane to be fully illuminated in succession and when this is accomplished may be found to be almost resting upon or looking over the opposite shoulder, so great are the curvatures in the vertical and horizontal planes of the meatus that sometimes hinder the view. Brunton's otoscope is a great favourite with some, but it is certainly a mistake to learn to make aural examinations with it alone to the exclusion of the forehead mirror and speculum, since the latter possess many advantages, chief amongst which is the facility they afford us for assisting our sense of sight by that of touch with the help of a probe held in the hand which is at liberty. Should a magnifying power be desired, a speculum can be employed with lens adapted, or if manipulations are unnecessary, Siegle's speculum answers extremely well, the glass window of the latter may be had either plane or spherical, and the instrument enables us to ascertain during inspection the mobility of the membrane and ossicles. (Fig. 10).

**Morbid Appearances of the Drum Membrane.**—The practitioner must not anticipate too much as regards the information he is likely to gain from observations of the drum membrane in chronic middle ear catarrh, and in order to place a right interpretation upon what he does see, it is at least necessary that he should be thoroughly familiar with the appearance of the membrane and its various surface markings in a state of health (figs. 4 and 5).

FIG. 4.—Normal membrana tympani of right ear. FIG. 5.—Normal membrana tympani of left ear.
Double size, from Politzer, edited by Dalby, 1894.

In estimating the degree of retraction present, note carefully the following points, which are usually to be seen in a well marked case. The handle of the malleus is foreshortened, and the short process white and prominent, like a little ivory knob with a thin membrane tightly stretched over it. The anterior and posterior folds look, as Politzer has stated, like tendinous grey bands, and Prussak's striæ, which converge from each extremity of the Rivinian segment to meet at the point of the short process—and enclose Schrapnell's membrane, are also unusually conspicuous. The cone of light is generally altered in shape, or may even be absent altogether, but its great liability to variation in a state of health renders it a less trustworthy index of pathological change. With much indrawing of the membrane, there is rarely a perfect state of polish and transparency, but should this condition obtain, the long process of the incus descending to articulate with the head of the stapes is unusually well seen, owing to the closer approximation of the membrane to it (fig. 6). The suffused dark

FIG. 6.—Retraction of membrana tympani, from Politzer, edited by Dalby. (Note promi ence of Rivinian striæ foreshortening of handle of malleus, and almost complete absence of bright spot.)

reddish lustre we sometimes see pervading the entire membrane in the first stage of a catarrh is due to the hyperæmic state of the epithelial investments of the inner wall of the tympanum rendered visible on account of the transparency of the membrane itself. Congestion of the drum head is characterised by injected capilliaries coursing around its circumference and down the handle of the malleus. A condition not very often seen, but which should always be looked for in recent catarrh, is that depending on the presence of serous fluid in the cavum tympani combined with a transparent state of the membrane (fig. 7).

The upper surface of the fluid is indicated by an appearance like that of a hair crossing the membrane horizontally and forming a concave, arched, or wavy line. A shake of the head alters its position, and frothy bubbles are seen when air is forced into the fluid through the Eustachian tube. Bullæ on the membrana tympani are usually caused by effusions of blood or serum beneath its epidermic layer, from a

Fig. 7.—Accumulation of effusion in inferior portion of tympanic cavity, from Politzer, edited by Dalby.

Fig. 8.—Semilunar chalky deposit in front of handle of malleus, from Politzer, edited by Dalby.

superficial myringitis, but bulging of the entire membrane in its posterior quadrant by fluid secretions have been seen. Thickenings and opacities, partial or general, are met with in the later stages of middle ear catarrh. In very old standing cases the entire membrane often has a white aud porcelain-like appearance. Calcareous deposits are distinct from simple thickenings, and result from a calcification of old inflammatory products within its layers (fig. 8). Another late condition is thinning and atrophy of the membrane resulting from absorption. This also may be strictly localized in spots, or may involve a considerable area in front of or behind the manubrium. Small patches of atrophy look like the cicatrices of old perforations (which, it should be remembered, have as in the case with Schrapnell' membrane, a cutaneous and mucous layer only without an intervening substantia propria); but they are not so well defined or deeply shaded. Thinned portions of membrane in the posterior quadrant when depressed, often allow the promontory or the incudo-stapedial joint to be seen through them. They bulge out on inflation by Valsalva or Politzer's method.

**Siegle's Pneumatic Speculum.**—We can judge best of the state of tension of the membrane, and the mobility of the ossicles as already observed, by the use of Siegle's speculum (fig. 10). Care must be taken to screw on a nozzle that is adapted to the size of the meatus, and to cover it with a piece of indiarubber tubing, so as to fix it hermetically without giving pain. When the air in the meatus is alternately compressed and rarefied by this means, atrophic portions are seen to flap in and outwards like the membrana flaccida, unless they are bound by adhesions to the inner wall. As regards the handle of the malleus, we infer if it oscillates with tolerable freedom, that there is at least no anchylosis of the malleo-incudal articulation. This is probably the most that can be said, sundry adhesions occasioning serious impairment of hearing are quite compatible with considerable movement of the membrane and handle of the malleus.

Fig. 10.—Siegle's Pneumatic Speculum.

It may be mentioned here that fixation of the stapes is said to be indicated, when with considerable deafness, there is no improvement in the hearing on being spoken to through a speaking trumpet (Gruber). There are no means at present known of diagnosing ankylosis of the stapes by inspection through the meatus.

**Testing the Patency of the Eustachian Tubes.**—Having carefully inspected the condition of the nose, throat, and nasopharynx, we next direct our attention to the condition of the Eustachian tubes, with a view to determining whether the case is one of simple and recent Eustachian catarrh, or if of longer standing, how much of the deafness is likely to be permanent. In the former case we are sure to find most of the signs of nasal catarrh still present from one or other of the causes that have been already mentioned, and we proceed to ascertain the results of inflation with Politzer's bag. This instrument is of paramount importance in the diagnosis and prognosis of chronic catarrh, and is our main reliance in treatment. If our case is one of quite recent origin and due to simple Eustachian obstruction, the patient will experience sudden relief on inflation, and regain his hearing power completely. Unfortunately this is not such a common experience as it ought to be, since we are too infrequently consulted at this stage.

**Politzer's Method.**—Politzeration (see fig. 11) is a simple enough procedure. The patient is seated opposite, with his head supported in a high-backed chair. Inserting one end of a diagnostic tube into our right ear and the other into the affected ear of the patient, we desire him to take a little water in his mouth. An air balloon, on the nozzle of which is fitted a soft india-rubber tip, having been selected, we insert the latter

Fig. 11.—Politzerization from Politzer, translated by Sir W. D alby, 1894

into the nostril—preferably the opposite one from the side in which the diagnostic tube is attached. Closing the alæ over the rubber tip with the finger and thumb of the left hand holding a tongue cloth, we desire the patient to swallow, and compress the bag suddenly and forcibly in the palm of the right hand at the moment the prominence of the thyroid cartilage is seen to rise in the neck. By requesting the patient or an assistant to firmly close with his finger the meatus of the opposite side we increase the pressure of the air current in the Eustachian tube of the ear under examination (Dundas Grant). Should a perforation exist in the membrane of the opposite side this precaution is absolutely necessary.

If the deafness is bilateral the auscultation tube is now transferred to the other ear, and the opposite meatus is closed in the same manner. It requires some experience to differentiate the sound which is referable to the air entering the Eustachian tube and tympanum from other sounds produced by the inflation. The greater part of the gurgling or thud heard by the unaided ear during Politzerization is due to the vibration of the soft palate which, by the act of swallowing, has been approximated to the posterior wall of the pharynx. The nasopharynx is most effectually closed and the tubal orifices rendered patent by the muscular contractions of deglutition; but for careless patients who splutter the water over the operator it is necessary to employ some modification, and we may direct them to blow out and fully distend their cheeks instead of swallowing water. Children are easily inflated whilst talking or crying. Politzerization should be employed in the first instance in every case, and the result noted. If it prove ineffectual, or should we desire to investigate more carefully the condition of the Eustachian tubes with reference to dryness or moisture, stenosis, &c., we must have recourse to catheterization.

Semi-diagramatic representation of tensor tympani, showing insertion into handle of malleus. Omitted from Article I (enlarged from Hartmann).

(*To be continued.*)

## Figures and Tables

**Fig. 4. Fig. 5. f1:**
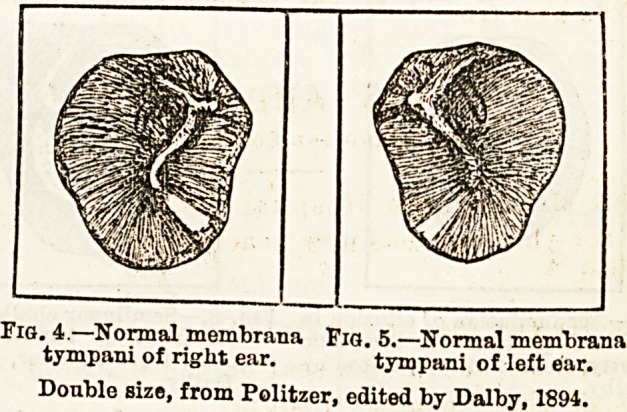


**Fig. 6. f2:**
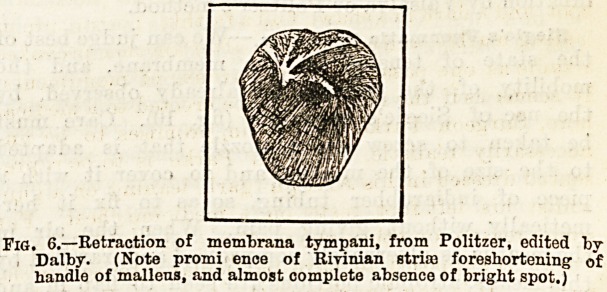


**Fig. 7. f3:**
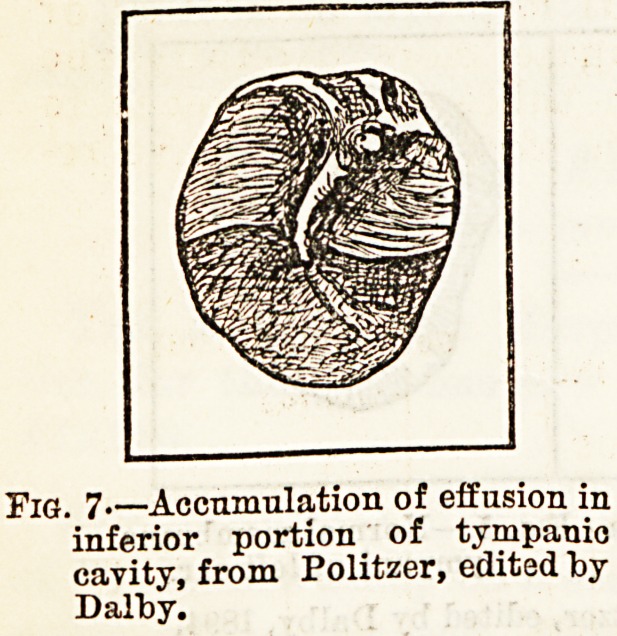


**Fig. 8. f4:**
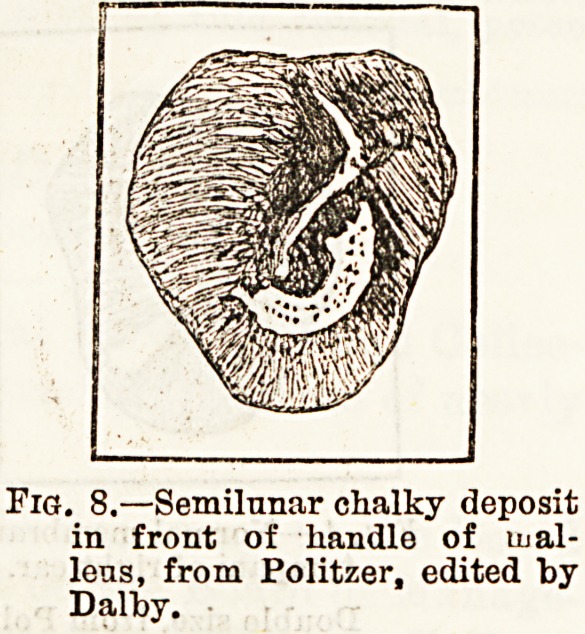


**Fig. 10. f5:**
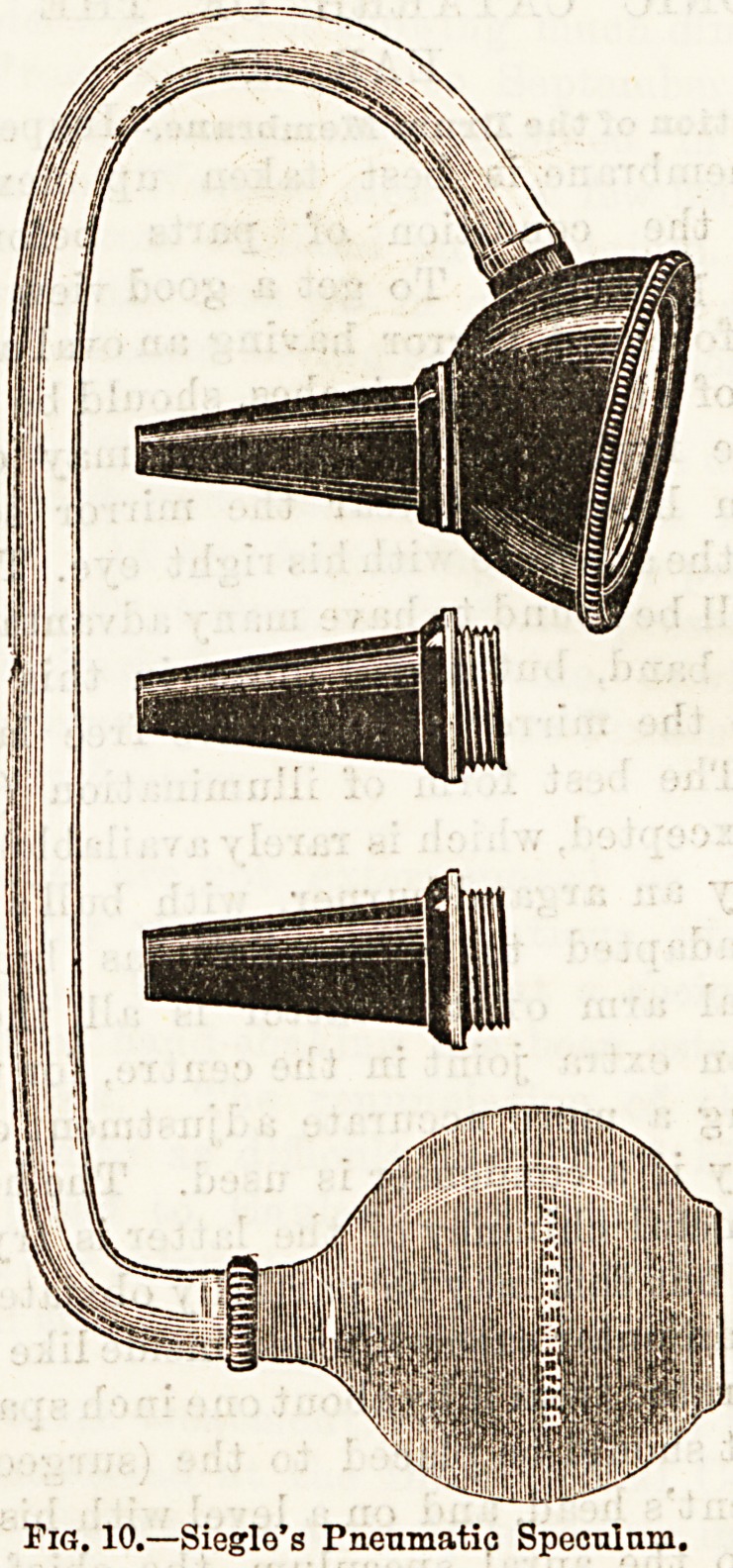


**Fig. 11. f6:**
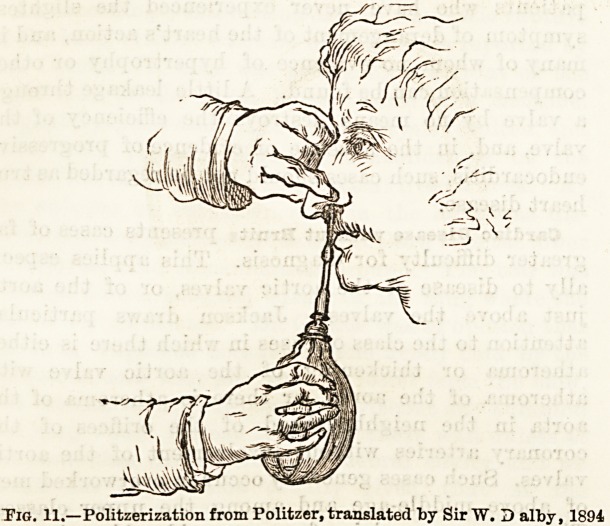


**Figure f7:**